# The wanderlust of a gallstone: a case report of intrathoracic migration of a gallstone post complicated cholecystectomy mimicking lung cancer

**DOI:** 10.1259/bjrcr.20150430

**Published:** 2016-11-02

**Authors:** Alya Saeed Binmahfouz, Karin Steinke

**Affiliations:** ^1^Department of Medical Imaging, Royal Brisbane and Women’s Hospital, Brisbane, QLD, Australia; ^2^School of medicine, The University of Queensland, Brisbane, QLD, Australia

## Abstract

Gallstones migrating into the right hemithorax post complicated cholecystectomy may be misdiagnosed for lung cancer, especially in the context of a distant history of cholecystectomy, poor recall of medical history and incomplete patient data. We present a case of a female patient with heavy smoking history who presented to our emergency department with haemoptysis and mild weight loss. Imaging workup showed an ^18^F-fludeoxyglucose positron emission tomography-positive heterogeneous nodule with central calcification in the right lower lobe, carrying lung cancer as a differential diagnosis. The resected specimen revealed an inflammatory pseudomass formed around a gallstone. This case illustrates the importance of knowing the spectrum of clinical and radiological presentation of a gallstone migrating into the right hemithorax, in order to prompt appropriate management and prevent misdiagnosis and mistreatment.

## Case report

**Figure 1.**
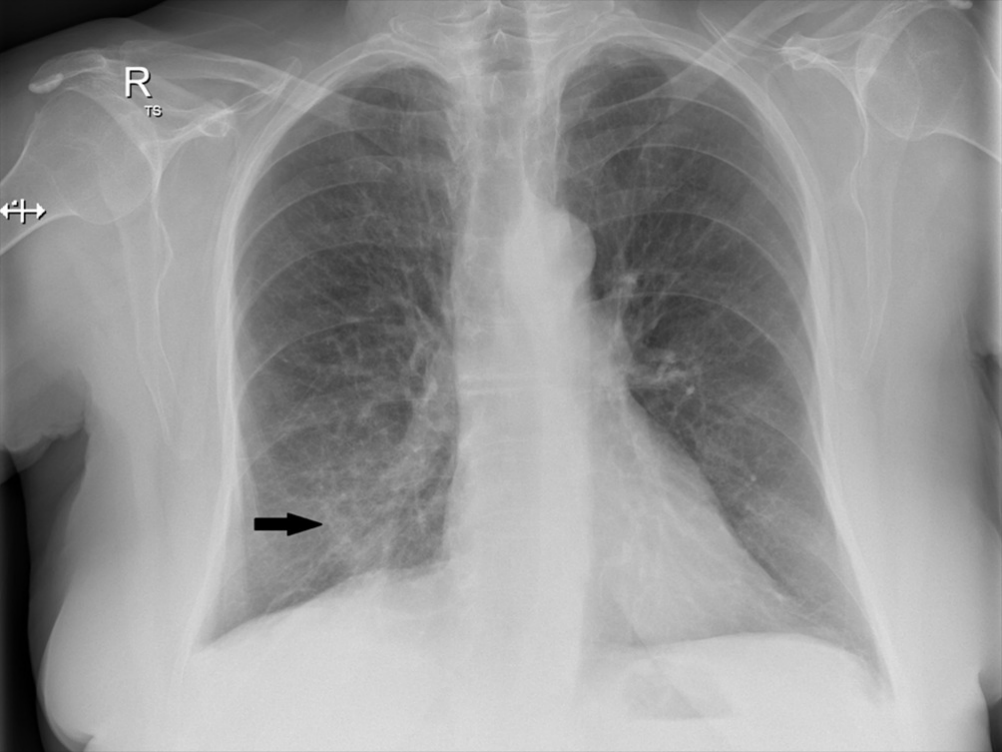
Frontal chest X-ray demonstrating a right lower lobe ill-defined opacity (arrow).

**Figure 2.**
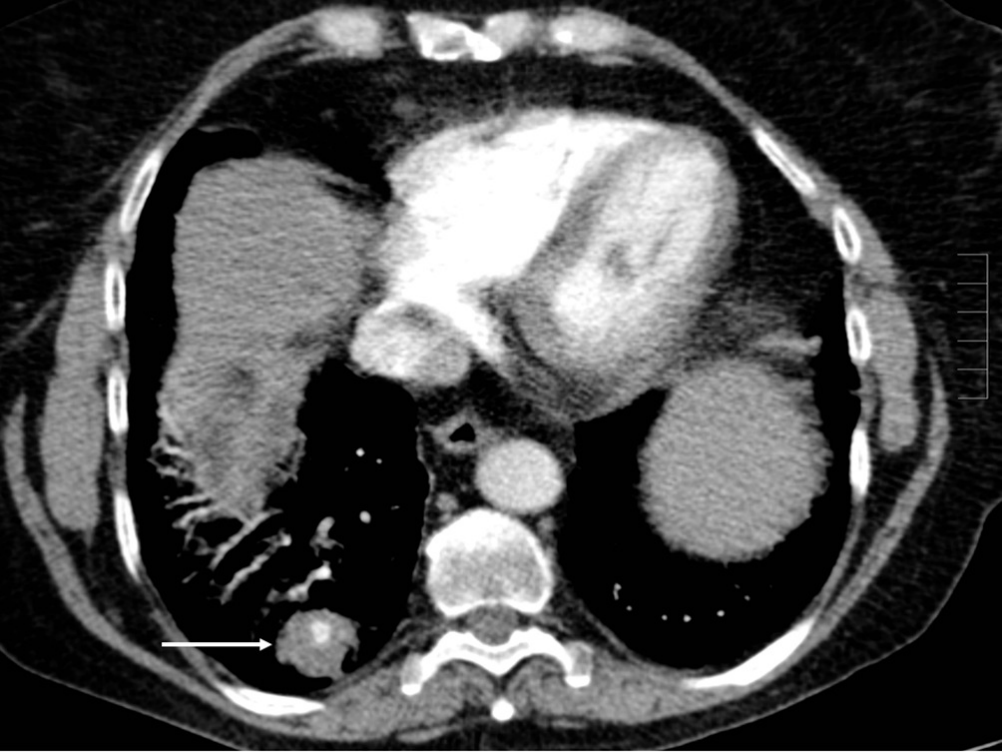
Axial contrast-enhanced CT scan of the chest in soft tissue window demonstrating a right lower lobe solid nodule (arrow) with central calcified focus.

**Figure 3.**
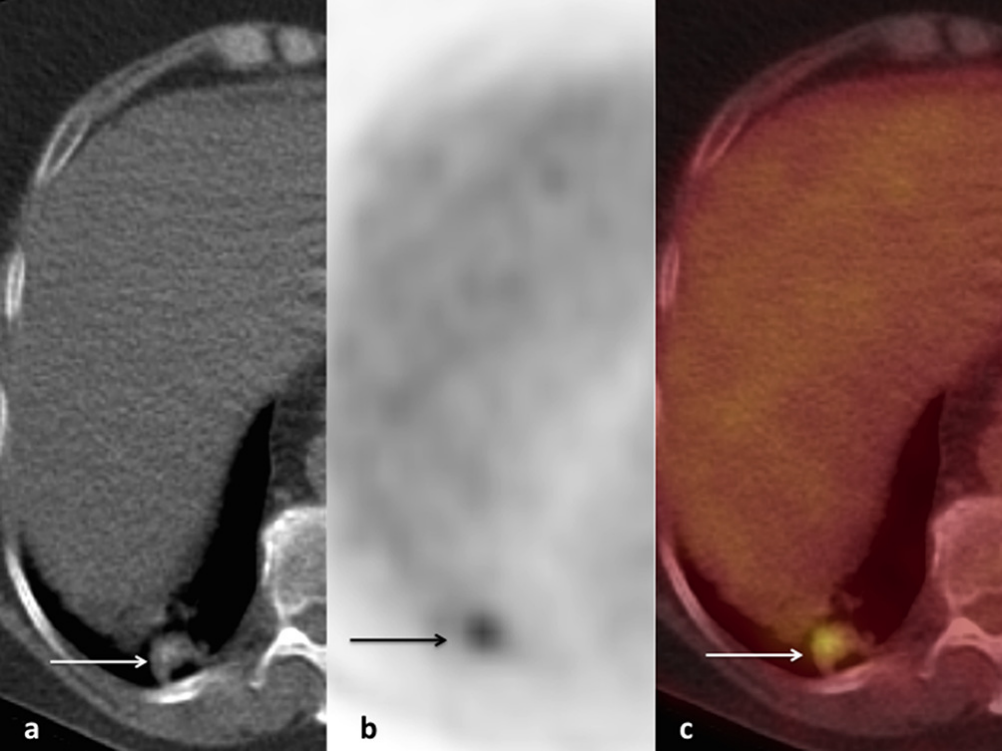
Axial non-contrast CT scan (a), FDG-PET scan (b), and fused PET/CT scan (c) showing focal increased FDG uptake within the right lower lobe nodule (arrows), maximum standardized uptake value of 5. FDG, ^18^F-fludeoxyglucose; PET, positron emission tomography.

A 66-year-old white female with a 50 pack-year smoking history presented to our tertiary hospital with acute massive haemoptysis. She had recently moved interstate, with no patient data available in our hospital system. The patient also reported anorexia and weight loss of approximately 3 kg in the past 6 months. On examination, she was afebrile, pulse rate was 90 beats min^–1^, respiratory rate was mildly increased at 22 breaths min^–1^ and blood pressure was slightly elevated at 150/90 mmHg. On auscultation of the chest, fine rales over the right lung base were noted. Laboratory results showed normal white blood cell count at 7300 cells l^–1^ (3.5–11 × 10^9^ cells l^–1^), C-reactive protein 14 mg l^–1^ (< 5 mg l^–1^) and haemoglobin 100 g l^–1^ (110–165 g l^–1^). Her initial chest X-ray showed an opacity in the right lower lobe (RLL) contiguous with the right hemidiaphragm (Figure 1). A CT pulmonary angiogram demonstrated a 2.8 cm solid enhancing nodule in the posterobasal segment of the RLL with a density measuring 35 HU, with a 7 mm central focus of dense calcification (Figure 2); also noted were hyperdense endobronchial material in the RLL, which was thought to represent fresh blood. A suspicion of lung cancer was raised, especially in view of heavy smoking history and reported weight loss. Positron emission tomography revealed increased ^18^F-fludeoxyglucose (FDG) uptake [maximum standardized uptake value (SUV_max_) of 5] within the RLL nodule; the report described the finding as concerning for malignancy, with the differential diagnosis of an inflammatory pseudotumour (Figure 3). A subsequent bronchoscopy was complicated owing to active bleeding from the RLL bronchus. The transbronchial biopsy showed no malignant cells, acid-fast bacilli or fungi.

**Figure 4.**
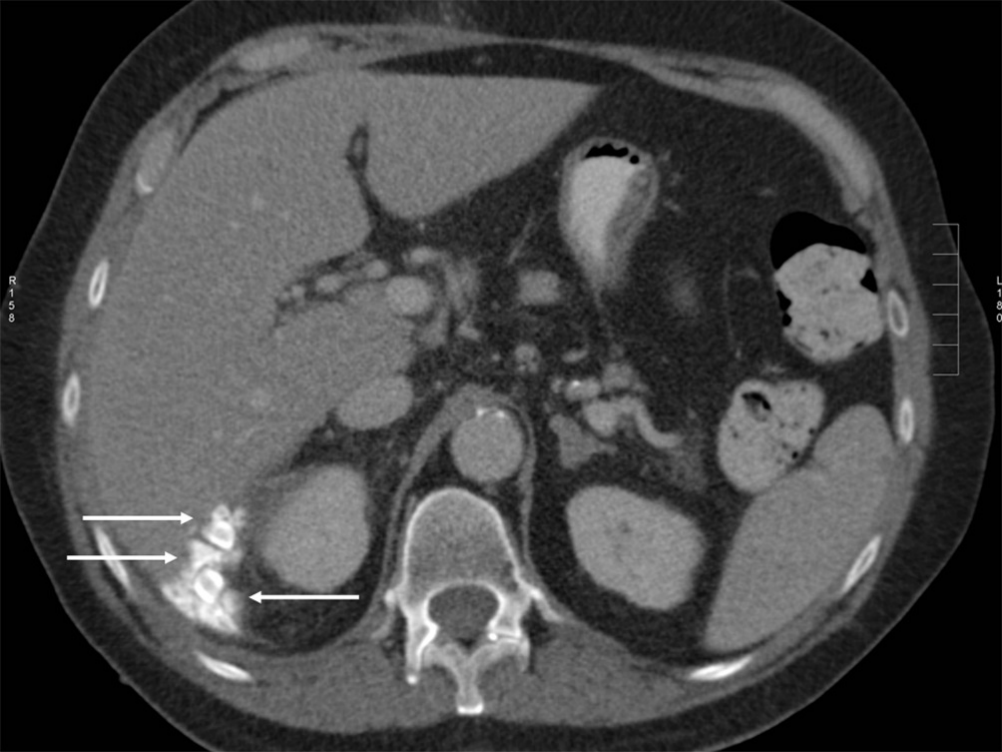
Axial contrast-enhanced CT scan of the abdomen demonstrating multiple spilt gallstones within Morison's pouch (arrows).

Upon targeted questioning, the patient disclosed having had a complicated cholecystectomy 3 years ago, performed at another hospital, with attempted laparoscopic cholecystectomy converted into an open laparotomy owing to gallbladder rupture with intraperitoneal spillage of gallstones; this was complicated by the formation of post-operative subphrenic abscess, which was surgically drained. The relevant externally performed images have been retrieved, including a CT scan of the abdomen (Figure 4). The patient further admitted to occasional episodes of minor haemoptysis of about two spoonfuls over the past 2 years, associated with right-sided mild chest pain, which she did not seek medical attention for.

**Figure 5.**
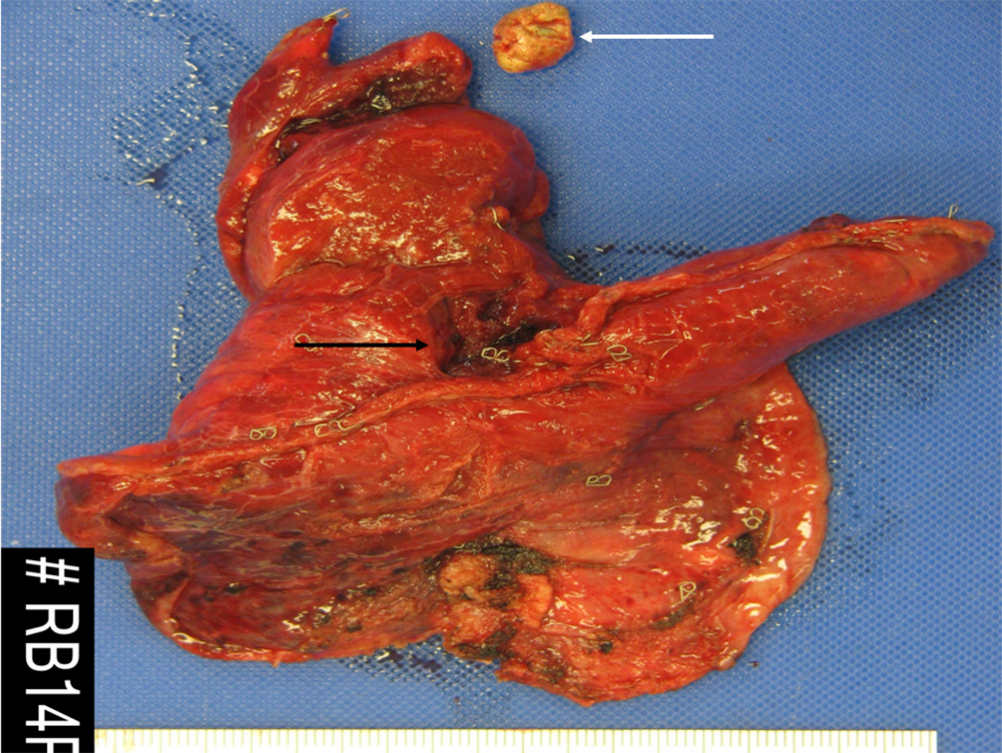
Fresh unopened lung wedge resection with staple line at the surgical margin, there is a defect on one side of this (black arrow). Separately received off-white to brown rhomboid stone (white arrow).

As the RLL mass was the presumed cause for the repeated episodes of haemoptysis, a thoracotomy was recommended and the patient underwent a RLL wedge resection. A firm rhomboid-shaped calculus measuring 11 × 7 × 8 mm (Figure 5) that dislodged from the specimen was confirmed to be a gallstone. Pathological examination additionally found abundant bile pigment (25%) surrounded by microorganisms, extensive interstitial fibrosis and hyalinization. Further biochemical analysis of the calculus revealed the presence of 85% cholesterol. Post-operative recovery was uneventful, and the patient was discharged from the hospital in a satisfactory condition 1 week after the surgery.

## Discussion

The incidence of gallstone spillage into the peritoneal cavity during laparoscopic cholecystectomy has been estimated to be between 0.2% and 32%, with a 6% incidence of conversion to laparotomy. The most common site of spilt gallstones is the right hypochondrium, accounting for 41% of cases.^1^

Migration of intrathoracic gallstones with inflammatory pseudotumour formation is very uncommon, but should be considered as a possibility in a patient post complicated cholecystectomy presenting with haemoptysis and a solid RLL lesion containing dense central foci of calcifications. A review of the literature shows a few reported cases of complicated laparoscopic cholecystectomy with intrathoracic gallstone migration.^2–5^ The time interval between clinical presentation, which includes haemoptysis, or less commonly cholelithoptysis, empyema or intrathoracic abscess, and surgery ranges from 2 to 60 months, with a mean of 12 months.^2^ By far, the most common thoracic site involved is the RLL, which is explained by the often subphrenic abscess post complicated cholecystectomy with direct transdiaphragmatic erosion owing to local inflammatory reaction.^2,3^ Furthermore, a study conducted by Guest^6^ explored the porous diaphragm syndrome that is responsible for conditions such as the predominantly right-sided peritoneal dialysis-related hydrothorax, bilious right-sided effusion with perforated gastric or duodenal ulcer and Meigs syndrome. Only one case of middle lobe involvement has been reported.^3^

Radiological investigations are the mainstay in the evaluation of haemoptysis, with causes including, but not limited to, acute infection, bronchitis, bronchiectasis, bronchogenic carcinoma and pulmonary embolism. However, at times, imaging can be equivocal and inconclusive owing to the non-specific findings of CT, as in our case where it showed a nodule in the RLL containing a central calcified focus. The differential diagnosis of a calcified pulmonary nodule/mass is unfortunately broad and includes calcified primary lung cancers; carcinoid; intrathoracic sarcomas; lung metastases primarily from sarcomas, gastrointestinal papillary and mucinous adenocarcinoma, and medullary carcinoma of the thyroid; and some benign lesions (hamartoma, calcified granuloma and amyloidoma).^7^

Moreover, FDG-positron emission tomography imaging can be misleading, as the increased metabolic activity can be due to inflammation, infection or malignancy. Furthermore, the SUV_max_ is also not helpful in narrowing down the differential diagnoses, as low-grade or well-differentiated tumours can have SUV values at or below background activity. In our case, the increased FDG uptake in the index lesion led to malignancy being mentioned as one of the differential diagnoses.

Conservative treatment with antibiotics has been reported, as well as spontaneous resolution.^1,2^ However, surgical approach is the mainstay of treatment and usually consists of wedge resection, anticipating an ectopic gallstone-related pseudotumour and aimed at removing the cause for the recurrent clinical symptoms rather than achieving clear margins, as would be the case with malignancy.

There is a reported case in the literature of a synchronous invasive adenocarcinoma, which had developed adjacent to a RLL gallstone-related pseudotumour. It is unclear if the cancer was a complication of the gallstone-incited chronic inflammatory changes or an unfortunate coincidental pathology.^8^

In conclusion, radiologists should be aware of the potential transdiaphragmatic migration of spilled gallstones post complicated laparoscopic cholecystectomy and the formation of an intrathoracic inflammatory pseudotumour.

## Learning points

1. Have a high index of suspicion for right lung base complications post complicated cholecystectomy with intraperitoneal spillage of gallstones.
2. Be familiar with the wide spectrum of clinical and radiological presentation of migrating gallstones.

## Consent

Written informed consent was obtained from the patient for publication of this report, including accompanying images.
